# A Novel Technique for Basilar Invagination Treatment in a Patient with Klippel–Feil Syndrome: A Clinical Example and Brief Literature Review

**DOI:** 10.3390/medicina60040616

**Published:** 2024-04-10

**Authors:** Masato Tanaka, Abd El Kader Al Askar, Chetan Kumawat, Shinya Arataki, Tadashi Komatsubara, Takuya Taoka, Koji Uotani, Yoshiaki Oda

**Affiliations:** 1 Department of Orthopaedic Surgery, Okayama Rosai Hospital, Okayama 702-8055, Japan; khattab2013@gmail.com (A.E.K.A.A.); dr.ckumawat@gmail.com (C.K.); araoyc@gmail.com (S.A.); t.komatsubara1982@gmail.com (T.K.); taokatakuya@gmail.com (T.T.); 2 Department of Orthopaedic Surgery, Okayama University Hospital, Okayama 700-8558, Japan; coji.uo@gmail.com (K.U.); odaaaaaaamn@yahoo.co.jp (Y.O.)

**Keywords:** basilar invagination, Klippel–Feil syndrome, navigation, C-arm free, novel technique

## Abstract

*Objectives and Background*: To present a novel technique of treatment for a patient with basilar invagination. Basilar invagination (BI) is a congenital condition that can compress the cervicomedullary junction, leading to neurological deficits. Severe cases require surgical intervention, but there is debate over the choice of approach. The anterior approach allows direct decompression but carries high complication rates, while the posterior approach provides indirect decompression and offers good stability with fewer complications. *Materials and Methods*: A 15-year-old boy with severe myelopathy presented to our hospital with neck pain, bilateral upper limb muscle weakness, and hand numbness persisting for 4 years. Additionally, he experienced increased numbness and gait disturbance three months before his visit. On examination, he exhibited hyperreflexia in both upper and lower limbs, muscle weakness in the bilateral upper limbs (MMT 4), bilateral hypoesthesia below the elbow and in both legs, mild urinary and bowel incontinence, and a spastic gait. Radiographs revealed severe basilar invagination (BI). Preoperative images showed severe BI and that the spinal cord was severely compressed with odontoid process. *Results*: The patient underwent posterior surgery with the C-arm free technique. All screws including occipital screws were inserted into the adequate position under navigation guidance. Reduction was achieved with skull rotation and distraction. A follow-up at one year showed the following results: Manual muscle testing results and sensory function tests showed almost full recovery, with bilateral arm recovery (MMT 5) and smooth walking. The cervical Japanese Orthopedic Association score of the patient improved from 9/17 to 16/17. Postoperative images showed excellent spinal cord decompression, and no major or severe complications had occurred. *Conclusions*: Basilar invagination alongside Klippel–Feil syndrome represents a relatively uncommon condition. Utilizing a posterior approach for treating reducible BI with a C-arm-free technique proved to be a safe method in addressing severe myelopathy. This novel navigation technique yields excellent outcomes for patients with BI.

## 1. Introduction

Klippel–Feil Syndrome (KFS) is an abnormal fusion of two or more vertebrae in the cervical spine caused by a failure in division or normal segmentation in early fetal development. It is believed that KFS occurs in 1 out of 42,000 births [1]. The clinical triad of KFS consists of a shortened neck leading to facial asymmetry, a low hairline, and restricted neck mobility. These characteristics were first described by Andre Klippel and Maurice Feil in 1912 [2]. Patients with KFS may have spinal stenosis, neurologic deficit, cervical spinal deformity, and instability. Patients with KFS are sometimes asymptomatic, however this instability may potentially lead to death [3].

**Figure 1:**
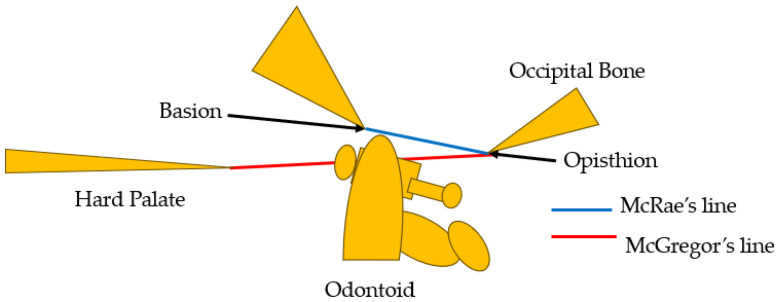
McGregor’s line [8] and McRae’s line [9].

Basilar impression was first reported by Ackermann in 1790 [4]. Basilar impression is characterized by odontoid displacement of the axis inwards towards the foramen magnum due to acquired softening of bones at the base of the skull, which can compress the cervicomedullary junction, causing neurologic deficit [5]. On the other hand, basilar invagination (BI) is defined as congenital upward displacement of vertebral elements into a normal foramen magnum with normal bone. The primary cause of BI is believed to be the presence of microtraumas resulting from repetitive lesions caused by instability [6]. In 1911, Schuller reported the radiological criteria for BI [7]. Diagnosis is currently made by observing protrusion of the odontoid over McGregor’s line [8] or McRae’s line [9]. McGregor’s line is defined as a line connecting the posterior edge of the hard palate to the most caudal point of the occipital curve. The diagnosis of BI is established when the tip of the dens lies more than 4.5 mm above this line [8]. McRae’s line, on the other hand, is a radiographic line drawn on a lateral skull radiograph. BI is diagnosed when the tip crosses this line [9] (Figure 1). The symptoms of BI are headache and/or neck pain, cranial nerve dysfunction, and quadriplegia [10].

The authors present the technical notes of a case involving a 15-year-old boy exhibiting symptoms attributed to basilar impression associated with Klippel–Feil syndrome. This study received approval from the ethics committee of our institute (No. 480), and necessary consents were obtained from the patient and his parents.

## 2. Case Presentation

### 2.1. Patient History

A 15-year-old boy with severe myelopathy was referred to our hospital. He had been experiencing neck pain, muscle weakness in both upper limbs, and numbness in both hands for 4 years. Increased numbness and gait disturbance emerged 3 months before his visit to our hospital. He is unable to run and has recently experienced dropping a cup several times.

### 2.2. Physical Examination

During the examination, he exhibited hyperreflexia in both upper and lower limbs and muscle weakness in both arms (MMT 4). Hypoesthesia was observed bilaterally below the elbows and in both legs. Additionally, he demonstrated clumsiness in both hands, mild urinary and bowel incontinence, and a spastic gait. His 10 s grip and release test yielded a score of 16 in both hands, with grip power measured at 20 kg in the right hand and 17 kg in the left. The cervical Japanese Orthopedic Association (JOA) score of the patient was 9/17.

### 2.3. Preoperative Imaging

**Figure 2:**
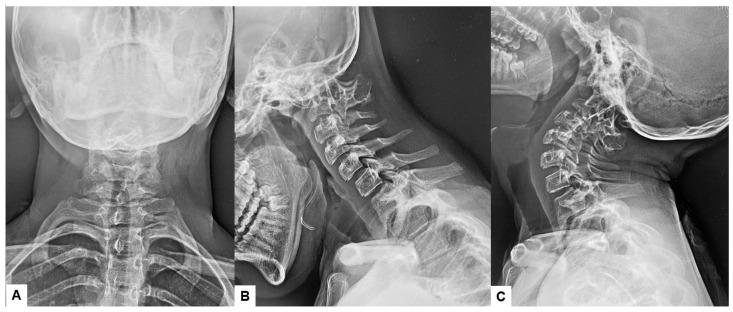
Preoperative radiograms, (**A**) Antero-posterior radiogram, (**B**) Lateral flexion radiogram, (**C**) Lateral extension radiogram. (**B**,**C**) show a C2/3 fusion anomaly. A dens protrusion into the foramen magnum measured 9.4 mm above McGregor’s line and 4.2 mm above McRae’s line.

**Figure 3:**
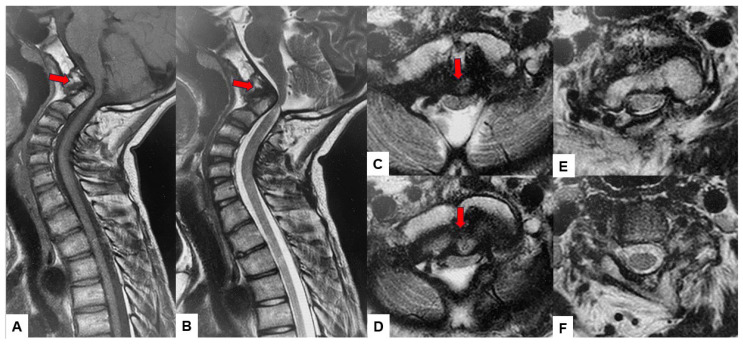
Preoperative MR imaging, (**A**) T1 weighted mid-sagittal MR imaging, (**B**) T2 weighted mid-sagittal MR imaging, (**C**) T2 weighted axial MR imaging at C1, (**D**) T2 weighted axial MR imaging at C1-2, (**E**) T2 weighted axial MR imaging at C2, (**F**) T2 weighted axial MR imaging at C3. The spinal cord was compressed severely due to basilar invagination. The red arrows show severe compression of the cervicomedullary cord by the dens.

Preoperative cervical radiographs revealed a short neck and a C2/3 fusion anomaly. Dens protrusion into the foramen magnum measured 9.4 mm above McGregor’s line and 4.2 mm above McRae’s line, with an anteroposterior (AP) diameter of the foramen magnum measuring 10.7 mm (Figure 2). Preoperative magnetic resonance imaging (MRI) depicted severe compression of the cervicomedullary cord by the dens, with a cervicomedullary angle (CMA) measuring 116 degrees (Figure 3). 

**Figure 4:**
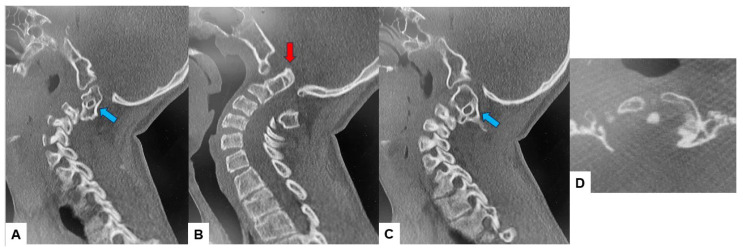
Preoperative CT, (**A**) Right sagittal reconstruction CT, (**B**) Mid-sagittal reconstruction CT, (**C**) Right sagittal reconstruction CT, (**D**) Axial CT at C1/2. The odontoid process was protruded into the foramen magnum (red arrow). C2 and C3 were fused (blue arrow).

**Figure 5:**
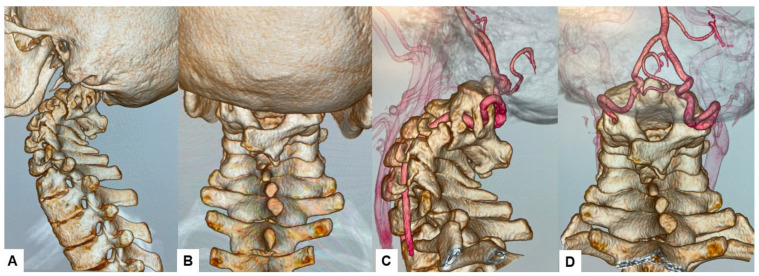
Preoperative 3D-CT and 3D-CT angiogram, (**A**) Lateral view 3D-CT, (**B**) Posterior view 3D CT, (**C**) Lateral view 3D-CT angiogram, (**D**) Posterior view 3D CT angiogram.

The CT scan clearly depicted the C2/3 fusion anomaly (Figure 4), while the 3D-CT scan revealed an abnormal course of the vertebral artery (Figure 5).

### 2.4. Surgery

**Figure 6:**
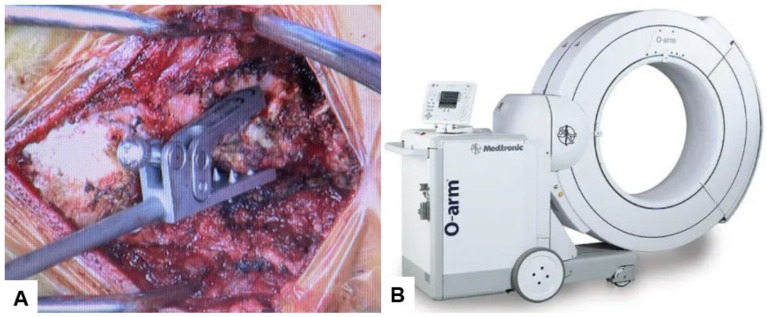
Reference frame and O-arm, (**A**) Reference frame, (**B**) O-arm.

The patient underwent posterior reduction with cervical pedicle screw fixation under the guidance of O-arm navigation, without a C-arm. The patient was positioned prone, with the neck in a neutral position on a Jackson frame equipped with a full carbon skull clamp to facilitate the O-arm scan. The procedure was conducted under neuromonitoring. The occiput and C1–5 were exposed with a 10 cm posterior midline incision. Initially, a reference frame was attached to the C2 spinous process (Figure 6).

**Figure 7:**
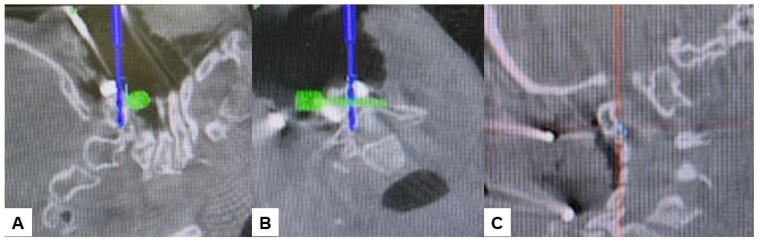
Bilateral C2 laminar screw, (**A**) sagittal view, (**B**) Axial view, (**C**) Oblique view.

**Figure 8:**
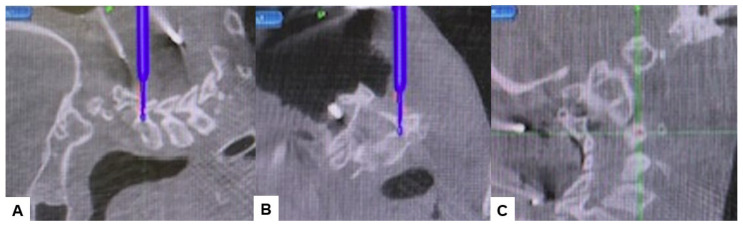
Pedicle screw fixation, (**A**) sagittal view, (**B**) Axial view, (**C**) Oblique view.

Subsequently, the O-arm was positioned, and three-dimensional (3-D) reconstruction images were obtained. Following the verification of each navigated mapped spinal instrument, bilateral C2 laminar screws (Figure 7) and C4–5 pedicle screws (Figure 8) were inserted under navigation. Pedicle screws were not inserted into the C2 vertebra because of bony anomaly and vertebral arteries course.

**Figure 9:**
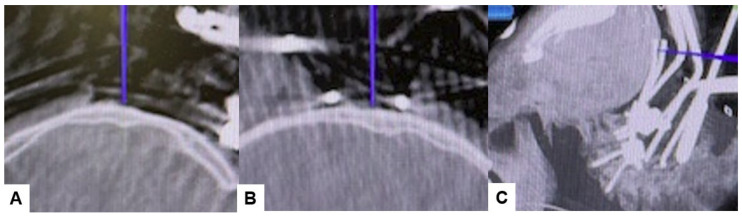
Occipital screwing, (**A**) sagittal view, (**B**) Axial view, (**C**) 3D view. The adequate screw point is indicated by the navigated pointer.

**Figure 10:**
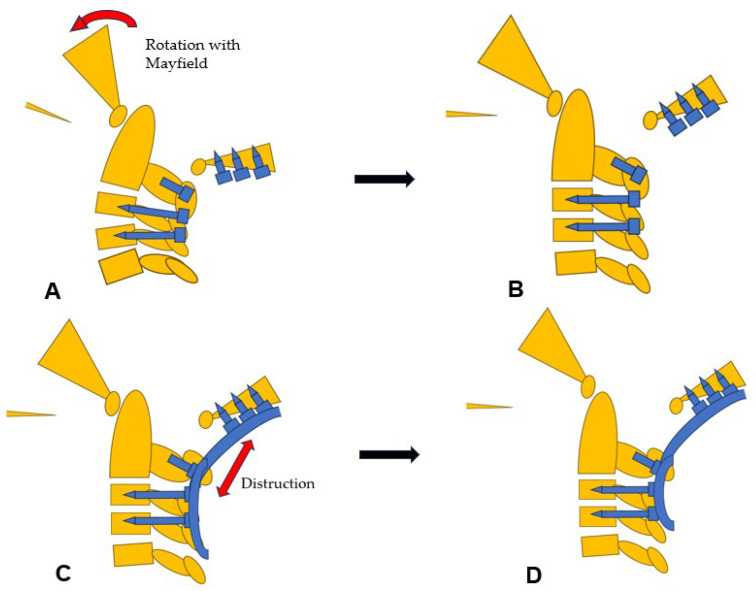
Reduction maneuver, (**A**) Before reduction, (**B**) Rotational reduction with Mayfield skull cramp rotation, (**C**) Distraction with screws and rods, (**D**) After reduction.

**Figure 11:**
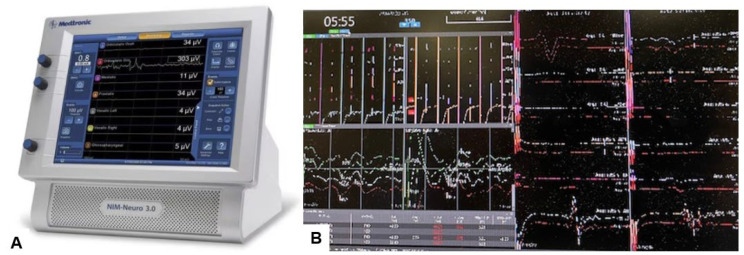
Neuromonitoring, (**A**) Intraoperative Neuromonitoring Systems (NIM™ 3.0), (**B**) Monitor image. Intraoperative neuromonitoring was used to prevent neurological deterioration during the reduction maneuver.

**Figure 12:**
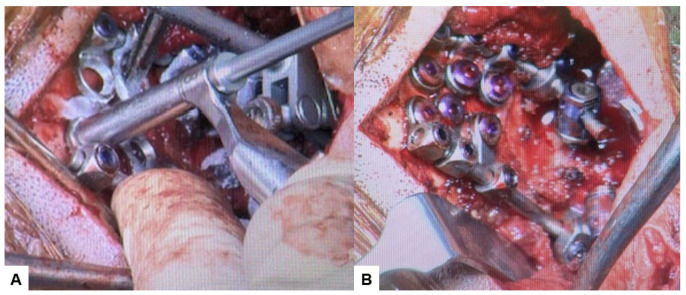
Intraoperative images, (**A**) Occipital screwing, (**B**) Rod insertion.

Then, under navigation guidance, the thickest portion for occiput screws was identified, and a total of 6 occipital screws were inserted using a navigated high-speed burr and pointer (Figure 9). The Mayfield skull clamp was loosened and the skull was rotated forward, with traction under neuromonitoring (Figure 10 and Figure 11). Finally, two cobalt–chrome rods were connected to the screw head and more distraction was performed with screw distraction for adequate reduction (Figure 12). 

### 2.5. Postoperative Imaging

**Figure 13:**
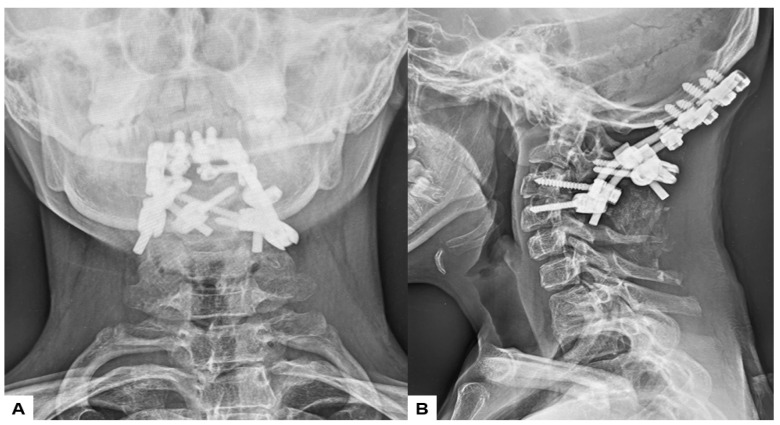
Postoperative radiograms, (**A**) Anteroposterior l radiogram, (**B**) Lateral radiogram.

**Figure 14:**
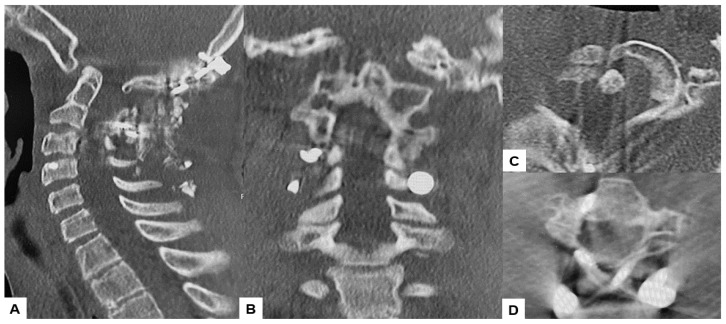
Postoperative CT. (**A**) Mid-sagittal reconstruction CT, (**B**) Coronal reconstruction CT, (**C**) Axial CT at C1/2, (**D**) Axial CT at C2/3.

Postoperative radiographs and CT scans demonstrated successful reduction, realignment, and appropriate screw positioning. The tip of the dens now measured 6.3 mm above McGregor’s line and 2.5 mm below McRae’s line, with the cervicomedullary angle (CMA) measuring 130 degrees. Additionally, the anteroposterior (AP) diameter of the foramen magnum increased to 19.3 mm (Figure 13 and Figure 14).

### 2.6. One Year Follow-Up

**Figure 15:**
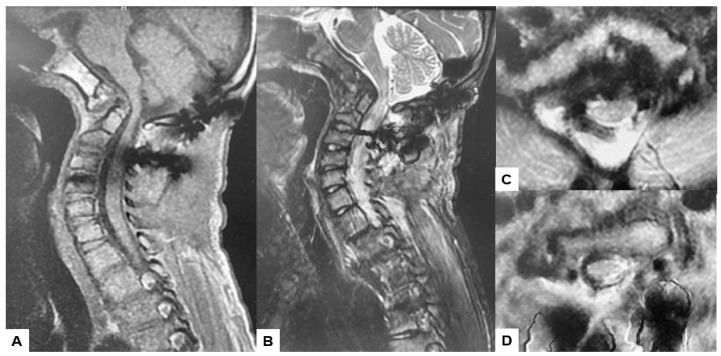
Follow-up images, (**A**) Mid sagittal T1-weighted MR imaging, (**B**) Mid sagittal T2-weighted MR imaging. (**C**) Axial T2-weighted MR imaging at C1, (**D**) Axial T2-weighted MR imaging at C2. The spinal cord was adequately decompressed.

Postoperative MRI indicated excellent spinal cord decompression (Figure 15). 

## 3. Results

Surgically, the patient was successfully treated, with a surgical time of 139 min and an estimated blood loss of 180 mL. During the one-year follow-up, manual muscle testing results and sensory function tests indicated almost full recovery in both bilateral arms (MMT 5). The patient is now walking smoothly without any gait disturbance, and the cervical Japanese Orthopedic Association score improved from 9/17 to 16/17. Postoperative radiographs demonstrated excellent spinal cord decompression, with no loss of reduction or malalignment. The cervicomedullary angle (CMA) postoperatively measured 130 degrees. Furthermore, there were no major or severe complications reported.

## 4. Discussion

Klippel–Feil syndrome is a complex condition mainly characterized by congenital malformation of the cervical spine where two or more vertebrae are fused. Patients typically present with radiculopathy and myelopathy, although instances of quadriparesis are infrequent [11,12]. These neurological symptoms are usually caused by spondylosis or instability of the adjacent segments to the fused vertebrae or by radicular compression within frequently undersized neuroforamina. Feil categorized Klippel–Feil Syndrome (KFS) into three types: Type 1 entails extensive fusion affecting multiple vertebrae, Type 2 entails fusion of two vertebrae, and Type 3 encompasses either of the other types combined with anomalies in the thoracic or lumbar spine [13]. The clinical presentation varies based on the extent and levels of fusion. Typically, fusions involving the cranio-cervical junction or extensive fusions are associated with earlier onset due to cosmetic deformity, pain, and delayed developmental milestones. Manifestation of lower cervical fusion often occurs later in life [14]. Type 2 patterns may typically be asymptomatic and reported as incidental findings on radiographic imaging, or when subaxial instability occurs, potentially leading to basilar impression, as observed in our patient’s case.

Basilar invagination (BI) refers to the migration or displacement of the odontoid in an upward direction, resulting in compression of the spinomedullary cord. The lower brain stem can be significantly affected by the dens, as it is positioned abnormally through the foramen magnum and into the posterior fossa [15]. Congenital basilar invagination may coincide with other abnormalities, such as atlanto-occipital fusion, atlas hypoplasia, hemirings of C1 with lateral mass spreading, odontoid abnormalities, Klippel–Feil Syndrome (KFS), and achondroplasia [16]. Suspecting basilar invagination is warranted when the C1–2 facet complex cannot be sufficiently visualized on a standard open-mouth anteroposterior view of the upper cervical spine [9]. Despite the wide use of plain radiographs with dynamic views as screening methods, MRI is still the best imaging modality for diagnosis because it shows how much neural impingement there is and the degree of cord compression [17]. CT angiography (CTA) is strongly advised preoperatively to detect any anomalous variations of the carotid and vertebral arteries, aiming to reduce the risk of intraoperative injury [18,19,20].

The use of traction with external fixation is considered in the treatment of BI, but this technique may benefit only a few patients without any neurological deficits [21]. Sekir recommended the utilization of traction. For the minority of patients without neurological disturbances, preoperative traction, both clinically and radiologically, for disease progression has been proposed as a viable alternative to operative stabilization [21,22]. In a case series by Goel et al., 82 patients without any associated Chiari malformation underwent cervical traction, leading to quick clinical improvement in 82% of these individuals after traction application [23]. Given that the patients included in the aforementioned studies exhibited mild neurological symptoms, this method may not be dependable for patients with severe basilar invagination and accompanying neurological deficits. Nonetheless, external fixation methods such as the halo vest pose several challenges, including pin loosening and infection risks, incomplete cervical spine fixation, inability to prevent progressive deformity, and the potential for serious complications like pin over penetration [24]. Following the approach outlined by Abumi et al., we opted not to undertake traction and manual reduction preoperatively to mitigate the risk of complications associated with external fixation. Surgical intervention was determined as the appropriate course of action for the patient [25].

Surgical treatment options for basilar invagination (BI) encompass various approaches and techniques, yet ongoing debate surrounds the optimal timing and choice of approach [26]. Historically, Chamberlain reported suboccipital craniectomy with cervical laminectomy and dural opening in 1939 [27]. His concept was based on relieving the compression on the cervicomedullary junction. However, the morbidity and mortality in these patients with this technique remained high [28]. The treatment algorithm for craniocervical junction abnormalities is divided into reducible and irreducible groups [26]. For reducible ones, posterior fixation is recommended. Irreducible pathologies are further divided on the basis of site of compression. For ventral stable pathologies, a transoral direst decompression is recommended. For ventral unstable ones, a transoral decompression followed by posterior occipitocervical fixation is ideal [29]. Another classification of BI was proposed by Goel et al. in 1998 [30]. He divided basilar invagination into two groups on the basis of presence or absence of Chiari malformation. In group 1, there is invagination of the odontoid process into the foramen magnum and it indents into the brainstem. In group 2, the assembly of the odontoid process, anterior arch of the atlas, and superior clivus migration in unison results in a reduction of the posterior cranial fossa volume.

The anterior approach is typically favored in cases where the protrusion of the odontoid process is irreducible and brainstem compression is severe [26,31]. The anterior approach is notably demanding, involving a complex technique with significant complications such as a higher incidence of postoperative infection and respiratory tract disorders. Additionally, it entails increased invasiveness and poses challenges in achieving primary fixation, often necessitating posterior instrumentation in subsequent cases [32]. Furthermore, the learning curve of anterior decompression is very steep. Decompression and instrumentation after acceptable reduction with the posterior approach is feasible in many cases, where the lesion can be managed with less complications related to the anterior approach [33]. Recently, a new endonasal endoscopic approach to pathologies of the anterior craniocervical junction was reported [34]. This technique is supported by preliminary anatomical and clinical studies exploring the feasibility and usefulness of approaching many ventral pathologies of the craniocervical junction.

The posterior approach generally provides stable fixation without requiring supportive external fixation or secondary stabilization. Unlike the anterior approach, this allows for early mobilization [35]. One of the most severe complications for occipito-cervical (O-C) fusion is postoperative dysphagia/dysphonia [36]. Reintubation after OC fusion is sometimes very difficult and requires tracheotomy [37]. To prevent this complication, Izeki recommended that the OC2 angle should be fixed at least at more than the preoperative O-C2 angle in the neutral position [38]. Neuromonitoring is mandatory for performing posterior indirect decompression because intraoperative OC alignment change may cause neurological deterioration. In irreducible cases, additional anterior surgery is necessary alongside posterior fixation [26].

In our novel technique, we demonstrate the effectiveness of a C-arm-free approach utilizing the O-arm with navigation via the posterior approach, allowing for reduction, decompression, and fixation of C0, C2, C4, and C5. Postoperatively, follow-up revealed successful reduction and rigid fixation with smooth recovery, without any serious complications occurring. The advantages of our new technique are: (1) Occipital screws can be inserted the thickest part of the skull very precisely under navigation guidance; (2) Pedicle screws can be inserted even in congenial anomaly vertebrae; (3) The most important point is that there is no radiation hazard to the surgeons and surgical staff. It has been reported that the accuracy of screw placement in the cervical spine is enhanced by the O-arm [39]. Additionally, the safe performance of atlantoaxial fixation using the O-arm has been demonstrated by Wada et al. [40]. Changes in navigation accuracy may occur during surgery, particularly if the position of the reference frame is inadvertently altered, potentially impacting the procedure’s accuracy. Reviewing the literature (Table 1) [41,42,43,44], the main technique used for screw insertion is the free hand technique using C-arm fluoroscopy, though there is still a risk of mal-insertion or violating important vital structures using this technique [45,46,47]; although, to our knowledge, no other study has addressed occipital screw fixation under navigation. Van de Kelft et al. (2012) reported a pedicle screw violation rate of 2.5% using navigation in the cervical spine [47]. In contrast, free-hand and fluoroscopy-assisted techniques have been linked to significantly higher rates of incorrect pedicle screw placement, ranging from 15% to 40% [48,49]. Another drawback of the C-arm technique is the increased radiation exposure for both the surgical team and the patient compared to our C-arm-free approach, which minimizes exposure for all parties involved [50].

Positioning occipital screws in occipitocervical instability poses a significant challenge, particularly to precision. It is crucial to accurately identify the thickest part of the lower occiput to safely insert the screws without risking injury to surrounding anatomical structures or the dura, which could lead to cerebrospinal fluid (CSF) leakage (Figure 7). Successful placement of occipital screws necessitates a thorough understanding of bone anatomy and its relationship with neurovascular structures, the spinal canal, hypoglossal canal, vertebral arteries, and the jugular foramen [44]. Using our technique, utilizing a navigation-mapped high-speed burr and probe, we achieved precise insertion of occipital screws with optimal length by directly visualizing and identifying the thickest part of the occiput. This approach, guided by navigation, ensures high accuracy and enhances screw purchase and strength.

This study has several limitations, including a small sample size, short follow-up duration, lack of a control group, and the need for statistical assessment of patient outcomes and complications with a larger population. A comparative study comparing navigational support to current methods of treating BI is warranted to further evaluate the efficacy of this technique.

## 5. Conclusions

Basilar invagination (BI) occurring alongside Klippel–Feil syndrome is a relatively uncommon occurrence. Utilizing a C-arm-free navigation technique for posterior reduction, indirect decompression, and fusion under neuromonitoring proves to be a safe approach in addressing this condition. The OC2 angle should be fixed at least at more than the preoperative O-C2 angle in the neutral position. This innovative method yields favorable outcomes for individuals with BI and a reducible odontoid.

## Figures and Tables

**Table 1:** Cranial screw position, lengths, and diameters.

Author	Safe Permissible Sagittal Plane Angulation (Degrees)	Medial Plane Angulation (Degrees)	Screw Length (mm)	Screw Diameter (mm)
La Marca et al. [41]	30 caudal	10 medial	22 (intraosseous)	3.5
Uribe et al. [42]	Zero to 5 cranial	15 medial	20 (intraosseous)	3.5
El-Gaidi et al. [43]	4 ± 6.2 caudad angulation (range, from 5 cranially to 12 caudally)	30 ± 6.7 (range, 20–40) medial	22 ± 3.1 (intraosseous)	3.5
Bosco et al. [44]	From 0 to 5 cranial	23–38 medial	19.9 ± 2.3 (intraosseous)	3.5

## Data Availability

The data presented in this study are available in the article.
